# Efficacy of extracorporeal shockwave lithotripsy with furosemide and hydration in renal stone management: A randomised controlled trial

**DOI:** 10.1080/2090598X.2019.1645262

**Published:** 2019-07-24

**Authors:** Safiullah Sohu, Munawar Hussain Soomro, Riaz Hussain Mangrio, Arif Ali Shaikh, Azizullah Mirani, Khoob Chand, Malik Hussain Jalbani

**Affiliations:** a Department of Urology, Chandka Medical College and Hospital, Shaheed Mohtarma Benazir Bhutto Medical University (SMBBMU), Larkana, Pakistan; b Pierre Louis Institute of Epidemiology and Public Health (IPLESP UMRS 1136), Saint-Antoine Medical School, Sorbonne Université and INSERM, Paris, France; c Department of Community Medicine, Al-Nafees Medical College and Hospital, Isra University-Islamabad Campus, Islamabad, Pakistan; d Department of Urology, Jinnah Sindh Medical University, Karachi, Pakistan

**Keywords:** Extracorporeal shockwave lithotripsy, furosemide, hydration, renal stone, Larkana

## Abstract

**Objective**: To assess the effect of diuretics (furosemide) administered before extracorporeal shockwave lithotripsy (ESWL) followed by continuous infusion of 0.9% NaCl during the ESWL in patients with renal stones.

**Patients and methods**: A tertiary care teaching hospital-based prospective randomised controlled trial was conducted from July 2015 to June 2017, including 714 patients who underwent ESWL. The patients were randomised in two groups: in Group-A, patients received 40 mg furosemide 30 min before each ESWL session and 1000 mL 0.9% NaCl intravenous hydration during the procedure. In Group-B, the patients only received 0.9% NaCl. All patients were followed-up every 2 weeks for 3 months with X-ray and ultrasonography of the kidney, ureter and bladder. Patients without a radio-opaque stone at follow-up were classified as successes.

**Results**: After 2 months, the stone-free rate (SFR) was much higher in Group-A, at 77.0% vs 65.3% (*P* < 0.001). Further, for patients aged ≤40 years, the SFR was significantly higher in Group-A than Group-B, at 89.2% vs 71.4% (*P* < 0.001). The mean (SD) age of the patients was 34.4 (8.23) years. Amongst them, 441 (61.8%) were male and 273 (38.2%) were female. The mean (SD) stone size was 1.42 (0.21) cm in Group-A and 1.40 (0.20) cm in Group-B.

**Conclusion**: We conclude that the efficacy of diuretics (furosemide) along with hydration is superior to hydration alone during ESWL for renal stone clearance.

**Abbreviations**: BMI: body mass index; KUB: kidney, ureter and bladder; OPD: Outpatient Department; ESWL: extracorporeal shockwave lithotripsy; SFR, stone-free rate.

## Introduction

Renal stones are one of the most prevalent urological health problems worldwide [1]. The incidence, prevalence and composition of the calculi vary according to geographic location. The prevalence ranges from 5% to 9% in Europe, 7–13% in North America, and 1–5% in Asia [2]. Such differences may involve several factors including: age, sex, fluid intake, dietary habits, occupation, educational level, race, socioeconomic status, and genetics. Due to excessive exposure to sunlight and high temperatures, a greater prevalence has been observed in South Asia and Southeast Asia, including Pakistan [3,4]. Furthermore, the stones located in different sites, i.e., upper or lower urinary tract, may have different compositions. Particularly in Pakistan, the dominant components of upper urinary tract calculi are calcium oxalate (75%) and hydroxyapatite (51%) [1,5].

The treatment options available for urolithiasis include; extracorporeal shockwave lithotripsy (ESWL), ureteroscopic stone extraction, percutaneous nephrolithotomy, medical therapy or a combination of treatment modalities [6]. However, in general ESWL, as minimally invasive procedure, is considered best for the management of urolithiasis in most patients, particularly when the stone size is <2 cm [7]. ESWL is a well-accepted first-line therapy for most renal stones in adults, as well as in the paediatric population [6,7]. Diuretics increase urinary flow around the stone during ESWL, which improves the likelihood of the cavitation phenomenon occurring. Diuretics are safe and if used adjuvant to ESWL, can provide a better outcome.

We hypothesised that the efficacy of diuretics (furosemide) along with hydration may be superior to hydration alone during ESWL for renal stone clearance. We assessed the effect of furosemide administered before ESWL followed by continuous infusion of 0.9% NaCl during the procedure in patients with renal stones.

## Patients and methods

A tertiary care teaching hospital-based prospective randomised controlled trial was conducted between July 2015 and June 2017 at the Department of Urology, Chandka Medical College Hospital affiliated with Shaheed Mohtarma Benazir Bhutto Medical University (SMBBMU), Larkana, Pakistan. Ethical approval was obtained as per protocol from the Ethical Review Committee (No. ERC/CMC/SMBBMU/1332) before the study.

All patients aged 18–50 years of either gender, with a single radio-opaque or non-opaque stone of ≤1.6 cm visiting the Department of Urology, were invited to participate in the study after a detailed informed consent. Patients with any anatomical abnormality, uncontrolled coagulopathy, untreated UTI, previous renal and/or ureteric surgery, ipsilateral ureteric stone, multiple or bilateral stones, congenital ureteric or renal abnormalities, renal insufficiency (serum creatinine >1.8 mg/dL), cardiac disease (previous cardiac failure, myocardial infarction, arrhythmia, cardiac surgery or ischaemic cardiac disease), known hypertensive or morbidly obese (body mass index [BMI] >40 kg/m^2^), were excluded. Additionally, patients with history of allergy to furosemide and pregnant women were also excluded.

### Data collection and analysis procedure

A detailed history; demographic characteristics and complete physical examination; basic laboratory investigations, including complete blood chemistry, urine analysis, urine culture and sensitivity (where indicated), blood urea, serum creatinine; ultrasonography and X-ray of the kidney, ureter and bladder (KUB) and IVU, were performed in every patient. All patients that met the eligibility criteria were selected. Patients were informed in detail about the advantages and disadvantages of the study and written consent was taken from all participants. Where the urine culture was positive, appropriate antibiotic therapy, according to the antibiogram, was given for an appropriate duration and ESWL was started after urine cultures were negative. The patients’ sex and age, location of the stone, maximum and mean densities and distance from the skin, number of ESWL sessions, number of pulses/session, total energy, effectiveness at 3 months after the final session, and complications were recorded.

A double-blinded randomisation (patients and physicians providing treatment) was carried out and the patients were divided into two groups, i.e., Group-A (furosemide + hydration) and Group-B (hydration only), using computer-generated numbers. A standard ESWL protocol was used for the 357 patients in each group. The patients in Group-A, received the standard ESWL protocol and additionally, 40 mg furosemide before each ESWL session and 1000 mL 0.9% NaCl i.v. hydration at 16.5 mL/min during the procedure. The patients in Group-B only received 1000 mL 0.9% NaCl during the procedure. Blood pressure monitoring was also performed for all patients during the procedure. All patients were treated with no general or spinal anaesthesia.

A Dornier Compact Sigma ESWL machine (Dornier MedTech, Munich, Germany) was used, with manual operation, having both ultrasonographic and fluoroscopic support. About 3000 shockwaves at 60 shockwaves/min were given to each patient in a single sitting with a voltage-ramping treatment regimen (12 kV followed by 24 kV), which can significantly reduce tissue injury and facilitates better stone fragmentation. A single dose of Ceftriaxone (1 g, i.v.) was given 30 min before ESWL; and after ESWL, oral ciprofloxacin 500 mg twice daily and oral diclofenac 50 mg twice daily were given for 7 days. Patients were followed-up at the Outpatient Department (OPD) at 2 weeks after ESWL, the degree of fragmentation of the stone and residual pieces were assessed by comparing post- and pre-ESWL X-ray KUB. If evidence of a residual stone of >0.5 cm was found a second session of ESWL was given, with a maximum of three sittings. Those patients found with residual stones were classified as ‘ESWL resistant’. All patients were advised to report immediately to our OPD or Emergency Room, if any complication (e.g., fever with chills, burning micturition, haematuria, and colic) arose after the procedure.

All patients underwent follow-up every 2 weeks for 3 months with X-ray and ultrasonography KUB. Patients were also assessed for fever, tenderness, pain and/or haematuria. Laboratory investigations in the form of urine analysis, culture and sensitivity tests, blood urea, and serum creatinine were repeated, if required. Patients with absence of radio-opaque shadow were classified as successes. The final result was assessed after 3 months.

The mean ± SD of numeric response variables, such as age and stone size, were assessed. The Student’s *t*-test was used for comparing stone clearance, i.e., stone-free rate (SFR). Percentages and frequencies were assessed for categorical variables, such as gender, SFR, and efficacy. Data were double entered in EpiData software version 3.1 (The EpiData Association, Odense, Denmark) and was analysed by using the Statistical Package for the Social Sciences (SPSS®) version 20.0 (SPSS Inc., IBM Corp., Armonk, NY, USA). Further stratification was carried out to control for the effects of modifiers, such as age and gender, the chi-squared test was applied considering a *P *< 0.05 as statistically significant.

## Results

**Figure 1.**
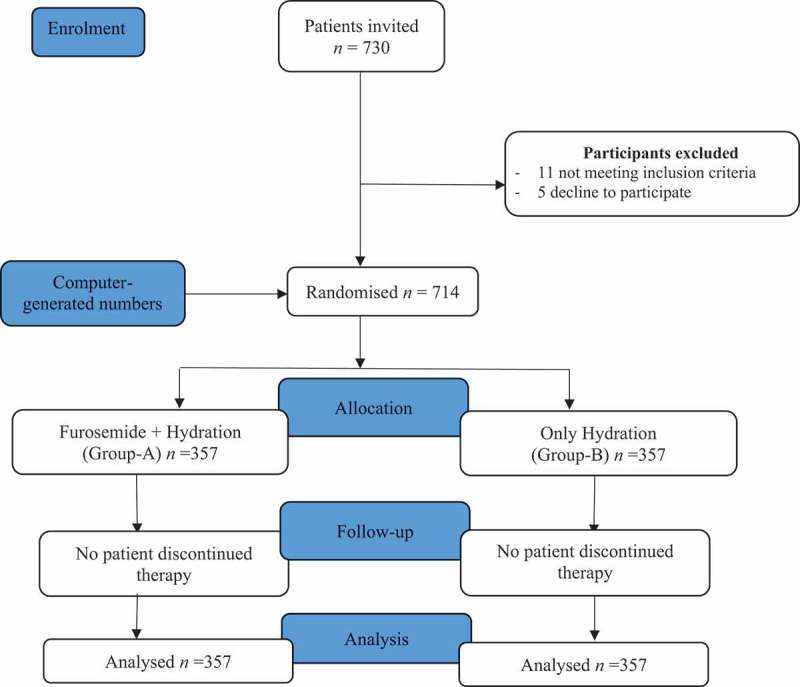
Trial flow chart.

Of a total of 730 patients, 16 were excluded due to either their not meeting the inclusion criteria or declining to participate. Hence, 714 patients were included, with 357 patients randomly allocated in each arm (Figure 1).

Of the 714 patients, 67 (18.7%) and 66 (18.4%) were aged ≤25 years in Group-A and Group-B, respectively; whilst 97 (27.1%) and 84 (23.5%) were respectively aged >40 years (Table 1). The patients’ mean (SD) age was 34.4 (8.23) years. There were 231 (64.71%) males in Group-A and 210 (58.82%) in Group-B. The mean (SD) stone size was 1.42 (0.21) cm in Group-A and 1.40 (0.20) cm in Group-B. Regarding stone location, 239 (61.34%) and 164 (45.94%) were located on the left-side and 138 (38.68%) and 193 (54.05%) were located on the right-side in Group-A and Group-B, respectively. The mean (SD) total number of shocks was 3147 (2131) in Group-A and 3286 (2277) in Group-B, and the total number of sessions was respectively, 2.17 (1.19) and 2.31 (1.27).

**Table 1.** Demographic characteristics of the study population.

Characteristic	Group-A *N* = 357	Group-B *N* = 357	*P*
Age, years, mean (SD)	34.79 (8.67)	34.02 (7.76)	
Age (years), *n* (%)			
≤25	67 (18.77)	66 (18.49)	0.03
26–30	83 (23.25)	85 (23.81)	
31–35	30 (8.40)	56 (15.69)	
36–40	80 (22.41)	66 (18.49)	
>40	97 (27.17)	84 (23.53)	
Gender, *n* (%)			
Female	126 (35.29)	147 (41.18)	0.11
Male	231 (64.71)	210 (58.82)	
Side, *n* (%)			
Right	138 (38.66)	193 (54.06)	<0.001
Left	219 (61.34)	164 (45.94)	
Stone size, cm, mean (SD)	1.42 (0.21)	1.40(0.20)	0.41
Serum creatinine, mg/dL, mean (SD)	1.11 (0.18)	1.10(0.21)	0.32
Stone density, HU, mean (SD)	778.37 (224.66)	779.52 (219.41)	0.83
Skin to stone distance, cm, mean (SD)	9.98 (1.89)	9.95 (1.91)	0.74

At 2 months follow-up, we compared the efficacy in terms of SFR of ESWL with and without furosemide along with hydration for renal stone management (Table 2). Renal SFR was 275 (77.0%) and 233 (65.3%) in Group-A and Group-B, respectively, and was significantly higher in Group-A than Group-B (*P* < 0.001). We further stratified the patients according to age, i.e., ≤40 or >40 years. In this stratified analysis, the renal SFR was significantly higher in Group-A than Group-B for the patients aged ≤40 years (89.2% vs 71.4%, *P* < 0.001). However, there was no statistically significant difference between the two groups for patients aged >40 years.

**Table 2.** Outcomes of the study at 2 months.

Stone clearance at 2 months	Group-A (*n* = 357), *n* (%)	Group-B (*n* = 357), *n* (%)	Total, *N* (%)	*P*
All patients				
Yes	275(77.0)	233(65.3)	508(71.1)	<0.001
No	82(23)	124(34.7)	206(28.9)	
Female				
Yes	103(81.7)	93(63.3)	196(71.8)	<0.001
No	23(18.3)	54(36.7)	77(28.2)	
Male				
Yes	172(74.5)	140(66.7)	312(70.7)	0.072
No	59(25.5)	70(33.3)	129(29.3)	
Age ≤40 years				
Yes	232(89.2)	195(71.4)	427(80.1)	<0.001
No	28(10.8)	78(28.6)	106(19.9)	
Age >40 years				
Yes	43(44.3)	38(45.2)	81(44.8)	0.90
No	54(55.7)	46(54.8)	100(55.2)	
Total number of shocks, mean (SD)	3147 (2131)	3286 (2277)		0.22
Total number of sessions, mean (SD)	2.17 (1.19)	2.31 (1.27)		0.85

In female cases, the SFR was 81.7% in Group-A and 63.3% in Group-B, which was significantly different (*P* = 0.001). Amongst male cases, the SFR was 74.5% in Group-A and 66.7% in Group-B, which was not statistically significantly different.

There was no significant difference in the complication rates between the two groups. However, 17 patients in Group-A and 14 patients in Group-B developed Steinstrasse formation and were treated with anti-inflammatory and α-blocker drugs and observed for 3 weeks. Ureteric stricture was reported in four and five patients in Group-A and Group-B, respectively; and ureterorenoscopic balloon dilatation was performed in these patients. All other reported complications were mild and were managed conservatively.

## Discussion

We investigated the efficacy of diuretics (40 mg furosemide, i.v.) along with continuous hydration (0.9% NaCl) during ESWL, with the hypothesis that it may be superior to hydration alone during ESWL for renal stone clearance. We found that the furosemide with hydration during ESWL was superior to hydration alone for renal stone clearance. This supports previous clinical trials, which showed that diuretics given i.v. accompanied by fluid infusion during ESWL improves the success rate [8,9].

Renal stones are one of the common problems in Pakistan because of its geographical location. Pakistan lies in a ‘stone belt’ region, which extends from Egypt, Iran, India and Thailand to Indonesia and the Philippines [10]. The use of ESWL for treating patients with renal stones has brought about a revolution in the field of urology. The procedure is cost-effective, as well as reducing hospitalisation time and morbidity. About 12% of the population will have urinary stone disease during their lifetime and the recurrence rate reaches 50% [11].

In our present study, patients underwent ESWL for solitary renal stones and were aged 18–50 years, with a mean (SD) age of 34.4 (8.23) years. Most of the men had their first episode at the age of 30–40 years. Similar results have also reported in other studies from Pakistan [12,13]. In our present study, of 714 cases, 61.8% were male and 38.2% were female, which shows the predominance of the male gender. Studies from other parts of the world have also shown a high prevalence amongst males [12–14].

In our present study, the SFR was significantly better in Group-A as compared to Group-B (77.0% vs 65.3%, *P* < 0.001). A clinical trial comprised of 106 patients from Egypt [9] included patients who were infused with 500 mL saline containing 40 mg furosemide and compared its effect with that of standard ESWL for the treatment of ureteric stones at different levels. The study reported that the treatment group required fewer sessions and fewer shockwaves/stone, and fragmentation and success rates were better regardless of the stone’s location. The calculus fragmentation and 3-month SFR were 93.3% and 87.5%, respectively for ESWL with diuresis compared to 70.6% (for fragmentation) for ESWL without diuresis. However, a Turkish single-blinded randomised controlled trial [15], showed that the comparison of the treatment group with the control group had no statistically significant difference in the average number of sessions, number of pulses and total energy used; that is, no benefit was obtained with the additional treatment. In that study the SFR was 71% and 69% in the treatment group and control group, respectively. Another study also showed that the use of diuretics along with ESWL treatment of renal and upper ureteric calculi did not statistically significantly improve stone fragmentation or clearance [6]. Clearance was achieved in 77.1% of the patients in the furosemide arm vs 70.8% in the placebo arm.

The influence of diuretic therapy on the success rate of ESWL was also investigated by Zomorrodi et al. [8]. The standard ESWL protocol was used in a group of 43 patients, and another group of 43 patients received 40 mg furosemide before ESWL. The SFR was 68.2% and stone fragmentation rate was 81%, in the control group, but in the treatment group, the rates were 88.4% and 93.1%, respectively. Regardless of the location of the ureteric stones, they reported that the addition of the diuretic to ESWL therapy improved both stone fragmentation and SFRs. In another clinical trial, including 115 patients conducted by Jafri et al. [16], the effect of furosemide on the success rate of ESWL in patients with renal or ureteric stones was studied. The treatment group receiving diuretic had a SFR of 71.9% compared with 39.7% amongst the controls (*P* = 0.007). Whereas the success rate for those with renal stones was 63.3% in the treatment group and 43.8% in the controls, those with ureteric stones had a success rate of 81.5% vs 20%. The increase in success found in the furosemide-treated patients was seen particularly when their BMI was >30 kg/m^2^ (81.3% vs 38.9%).

Another study investigated the effects of diuretic therapy on renal stones treated with ESWL [17]. The study included 52 patients with renal stones <2 cm in diameter, randomised into two groups of 26, one group was given hydrochlorothiazide twice daily and the other group was given a placebo. This study showed that the diuretic regimen did not affect the SFR after 3 months, but it did reduce the number of ESWL sessions and increased the stone-free status duration.

Renal patients are usually in a state of relative dehydration, due to use of purgatives the night before, which improves its effectiveness and fasting until the next day. The dehydration caused by relative oliguria might have a negative effect on stone fragmentation and the fluid film surrounding the stone reportedly enables improved fragmentation of the stone [15]. So, if the treated kidney’s function has deteriorated due to the obstruction, the dehydration hinders adequate urine flow around the stone [18]. Diuretics will increase urine flow and thus the probability of cavitation [19,20]; which means, pieces of the broken stone shell increase, and the centre of the stone is exposed to subsequent shockwaves allowing entry of urine through the broken surface, thus giving an adequate interface with the centre of the stone [15]. The size of the stone is an important prognostic factor for successful ESWL and is considered as a highly effective treatment option for lower pole stones ≤2 cm [21].

### Strengths and limitations

Our present study has strengths, as well as some limitations. The main strengths of the present trial are its prospective randomised study design and large sample size. Further, it was conducted within the routine healthcare setting, using the same staff. It was designed and developed to be potentially replicable and sustainable in the routine system, and research protocols and tools were piloted before the trial. In addition, healthcare providers and patients were blinded to the treatment allocation, which is also an added strength of the present study. The study has several limitations. The study was conducted in only one centre; however, the study centre covers a large population, including other provinces. Further information regarding use of analgesics, chemical analysis of stones, return to normal daily life, and total cost analysis would have been useful and would have strengthened the study.

## Conclusion

We conclude that the efficacy of diuretics (furosemide) along with hydration is better than hydration alone during ESWL for renal stone clearance.
